# Highly blood perfused, highly metabolically active pancreatic islets may be more susceptible for immune attack

**DOI:** 10.14814/phy2.14444

**Published:** 2020-07-03

**Authors:** Sara Ullsten, Daniel Espes, My Quach, Malin Fex, Monica Sandberg, Per‐Ola Carlsson

**Affiliations:** ^1^ Department of Medical Cell Biology Uppsala University Uppsala Sweden; ^2^ Department of Medical Sciences Uppsala University Uppsala Sweden; ^3^ Department of Clinical Sciences Lund University Diabetes Center Lund University Lund Sweden

**Keywords:** blood flow, heterogeneity, insulitis, pancreatic islets, type 1 diabetes

## Abstract

Differences in pancreatic islet susceptibility during type 1 diabetes development may be explained by interislet variations. This study aimed to investigate if heterogeneities in vascular support and metabolic activity in rat and human islets may explain why some islets are attacked earlier than other islets. In rats, highly blood perfused islets were identified by injection of microspheres into the ascending aorta, whereas a combination of anterograde and retrograde injections of microspheres into pancreas was used to determine the islet vascular drainage system. Highly blood perfused islets had superior function and lower glucose threshold for insulin release when compared with other islets. These islets had a preferential direct venous drainage to the portal vein, whereas other islets mainly were incorporated into the exocrine capillary system. In BioBreeding rats, the hypothesis that islets with high islet blood perfusion was more prone to immune cell infiltration was investigated. Indeed, highly blood perfused islets were the first affected by the immune attack. In human subjects, differences in glucose threshold for insulin (C‐peptide) secretion was evaluated in individuals recently diagnosed for type 1 diabetes and compared to control subjects. A preferential loss of capacity for insulin release in response to low glucose concentrations was observed at debut of type 1 diabetes. Our study indicates that highly blood perfused islets with direct venous drainage and lower glucose threshold for insulin release are of great importance for normal glucose homeostasis. At the same time, these highly metabolically active islets were the primary target of the immune system.

## Graphical abstract

Highly blood perfused islets with their direct venous drainage and lower glucose threshold for insulin release seem to be of great importance for normal glucose homeostasis. At the same time, these highly metabolically active islets are the primary target of the immune system which may explain why some islets are attacked earlier than other islets during the development of type 1 diabetes.

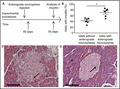

## 1 INTRODUCTION

There is a well‐known enigmatic heterogeneity of pancreatic islet pathology in the development of type 1 diabetes. Islets are not equally affected, where some islets and beta cells may be able to escape immune destruction for many years (Gianani et al., 2010; Keenan et al., 2010; Oram, Sims, & Evans‐Molina, 2019). In autoantibody‐positive subjects only 10% of islets showed immune cell infiltration before manifest diabetes onset, and in young subjects with recent onset diabetes infiltrating immune cells were present in 34% of the beta cell containing islets (In't, 2011; In't Veld et al., 2007). Although this variability in immune attack between islets could reflect accidental occurrence, there is also a possibility that it reflects anatomical, immunological or physiological differences. Heterogeneous expression of MHC class 1 between islets has been one suggestion to explain the heterogeneity in immune attack (Rodriguez‐Calvo et al.,^2015^).

Noteworthy, investigations of individual beta cells have also shown distinct populations with different functional, molecular, and morphological properties, including variability in cell maturation and glucose threshold (Bader et al., 2016; Gutierrez, Gromada, & Sussel, 2017; Pipeleers, 1987). There is recent evidence also indicating the existence of heterogeneity between islets within the healthy pancreas, including the presence of islets of different age with subsequent age related functionality of insulin secretion (Aguayo‐Mazzucato et al., 2017; Ellenbroek et al., 2013). We have previously reported on functional differences between islets based on their vascular support (Lau, Svensson, Grapensparr, Johansson, & Carlsson, 2012; Olsson & Carlsson, 2011; Ullsten, Lau, & Carlsson, 2015). In these studies, a high blood perfusion was coupled to a higher metabolic activity, a better glucose‐stimulated insulin release and an increased beta cell proliferation. Highly blood perfused islets were also more susceptible to cellular stress, and with an increased vulnerability for cellular death when exposed to hypoxia and cytokines (Lau et al., 2012; Ullsten et al., 2015).

In this study, we tested the hypothesis that heterogeneity in islet vascular support reflects anatomical and functional differences which correlate to susceptibility of the immune attack on the islets. To test our hypothesis, we performed studies in healthy rats, which were followed by studies in prediabetic BioBreeding (BB) rats, an animal model for type 1 diabetes, on human islets, and finally in individuals with recent onset type 1 diabetes.

## 2 MATERIALS AND METHODS

### 2.1 Experimental animals

Adult male Sprague–Dawley rats weighing 364 ± 9 g were purchased from Taconic M&B, whereas BB rats were obtained from a local colony bred at the Lund University (Regnell et al., 2017). The *lyp* region of diabetes‐prone BB rats was previously introgressed to the diabetes resistant (DR) BB rat. Heterozygote BB DR lyp/+ rats were kept in sibling breeding for more than 50 generations to yield 25% DR.lyp/lyp, 25% DR.+/+, and 50% DR.lyp/+ (MacMurray et al., 2002; Regnell et al., 2017). All experimental procedures were approved by the Animals Ethics Committee for Uppsala University.

### 2.2 Anterograde‐ and retrograde injection of microspheres

Rats were anaesthetized with 120 mg/kg body weight Inactin (thiobutabarbital sodium; Sigma‐Aldrich). Polyethylene catheters were inserted into the ascending aorta through the right carotid artery, and into the femoral artery. Colored (10 µm, E‐Z Trac Ultraspheres; IMT, Stason Labs) or fluorescent microsphere (10 μm, FluoSpheres Polystyrene Microspheres; Molecular Probes) identification of highly blood perfused islets was performed by injection into the ascending aorta through the catheter in the right carotid artery (Jansson & Hellerstrom, 1983). Free flow arterial blood sampling from the femoral artery started 5 s before microsphere injection of 2.5 × 10^5^ microspheres, diluted in 200 µl saline, and continued for a total of 60 s. This reference blood sample was used to calculate the blood volume each injected microsphere represented. The procedure of microsphere injection into the ascending aorta is denoted anterograde injection.

**FIGURE 1:**
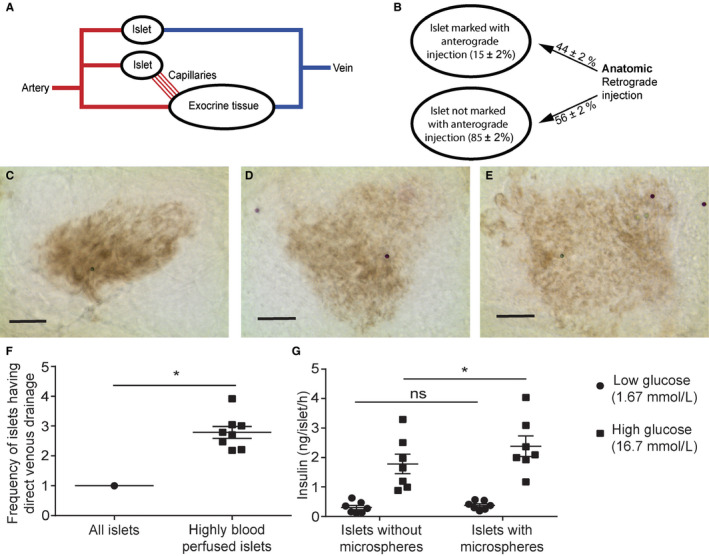
Characterization of rat islets with a direct venous drainage into the portal vein. (a) Schematic drawing of the drainage of pancreatic islets either directly into portal vein tributaries or into the capillary system of exocrine tissue. (b) Highly blood perfused islets, as identified by green microsphere content after anterograde injection, have a preferential venous drainage, as indicated by violet microsphere content after retrograde injection. Percentage values adjacent to arrows indicate the distribution of retrogradely injected microspheres. (c–e) Representative images of islets with microsphere content after (c) anterograde injection, (d) retrograde injection, and (e) both injections; scale bar 100 µm. (f) The frequency of highly blood perfused islets having a direct venous drainage is approximately three times higher than the mean frequency for all islets (data are expressed as fold increase, where the frequency of all islets having a direct venous drainage was set to 1). (g) Isolated islets with direct venous drainage, as identified by microspheres after retrograde injection, have a superior release capacity. Values are given as means ± *SEM*, *n* = 7–8, * denotes *p* < .05

In order to investigate the occurrence of direct venous drainage of islets, instead of drainage into capillaries in the exocrine parenchyma (cf. Figure 1a), also retrograde injections of microspheres (injections into the portal vein back toward the pancreas) were performed. When both injections were administered to the same animal, the anterograde injection was completed before surgical preparation for retrograde injection. For retrograde microsphere injection, the aorta was obstructed by a suture placed proximally of the junction of the celiac trunk. In order to avoid coagulation, ~10 µl of heparin (Heparin LEO, 5,000 IE/ml; Leo Pharma A/S) was injected into the femoral artery before this obstruction of normal blood circulation. Retrograde microsphere injection was performed through a polyethylene catheter inserted into the hepatic portal vein in the opposite direction of the normal blood flow. In order to avoid microsphere distribution into the vascular system of the lower gastrointestinal tract, the lower mesenteric artery was prior to injection ligated with a suture. Retrograde injection into the portal vein was performed with an injection speed of 100 µl/min during 5 min for a total injection volume of 500 µl with 2.8 × 10^6^ microspheres (diluted in saline). Free retrograde flow from the portal vein was obtained by an incision of the abdominal aorta distally of the suture placed proximally of the junction of the celiac trunk.

### 2.3 Calculation of blood flow

Since there is an upper limit on the amount of microspheres that can be injected anterogradely without causing hemodynamic disturbances, and since the mass of each pancreatic islet is so small, only islets with the highest blood perfusion will have microspheres after anterograde injection despite that the blood flow to the islet organ as a whole is known to be one of the highest in the body. For a detailed review of the possibilities, prerequisites, and limitations of the microsphere technique for measuring islet blood flow, see ref. Jansson et al. (2016). Interestingly, repeated microsphere injections indicate that the population of highly blood perfused islets does not change over time (Carlsson, Kallskog, Bodin, Andersson, & Jansson, 2002).

In order to exclude that the retrograde injection would remove microspheres already present in islets and exocrine pancreas after anterograde injection, islet and whole pancreatic blood flow was determined and compared between animals subjected to anterograde injection only and animals subjected to both anterograde and retrograde injection. The number of anterogradely injected microspheres was counted in the reference blood sample and in pancreatic endocrine and exocrine tissue after freeze‐thawing (Carlsson, Olsson, et al., 2002). Microspheres injected anterogradely could be distinguished from those injected retrogradely by the use of differently colored microspheres (green microspheres were used for anterograde injections and violet microspheres for retrograde injections). Blood flow was calculated by using the formula *Q*_org_ = *Q*_ref_ × *N*_org_/*N*_ref_, where *Q*_org_ is blood flow in the organ (ml/min), *Q*_ref_ is the blood flow in the reference sample (ml/min), *N*_org_ is the number of microspheres in the organ, and *N*_ref_ is the number of microspheres found in the reference blood sample.

### 2.4 Investigations of venous drainage

After both anterograde and retrograde injections of microspheres, the pancreas was removed and freeze‐thawed to visualize the islets (Carlsson, Olsson, et al., 2002). The number of islets with presence or absence of violet and green microspheres was counted in a microscope. The frequency of venous drainage (as identified by violet microspheres) of highly blood perfused islets (identified with green microspheres) was calculated as fold increase when compared with the frequency of venous drainage of all islets [fraction of islets with green and violet microspheres of all islets with green microspheres/fraction of islets with violet microspheres of all islets].

### 2.5 Islet isolation

Rat islets were isolated by collagenase‐digestion alone or in combination with a density gradient purification technique, hand‐picked and incubated for 3 days in groups of 100 at 37°C (Henriksnas et al., 2012; Sandler, Andersson, & Hellerstrom, 1987). Microsphere detection was performed in a fluorescence microscope and the islets were dichotomously sorted dependent on microsphere content (Lau et al., 2012).

### 2.6 Glucose‐stimulated insulin release

Groups of size‐matched 10 islets, with or without presence of microspheres after retrograde injection, were separately taken for analysis. The islets were firstly incubated in KRBH with a low glucose concentration (1.67 mmol/l) for 1 hr, and then in KRBH with a high glucose concentration (16.7 mmol/l) for a second hour. Released insulin during the incubations was analyzed by a Rat Insulin ELISA kit (Mercodia).

### 2.7 Glucose responsiveness by perifusion

**FIGURE 2:**
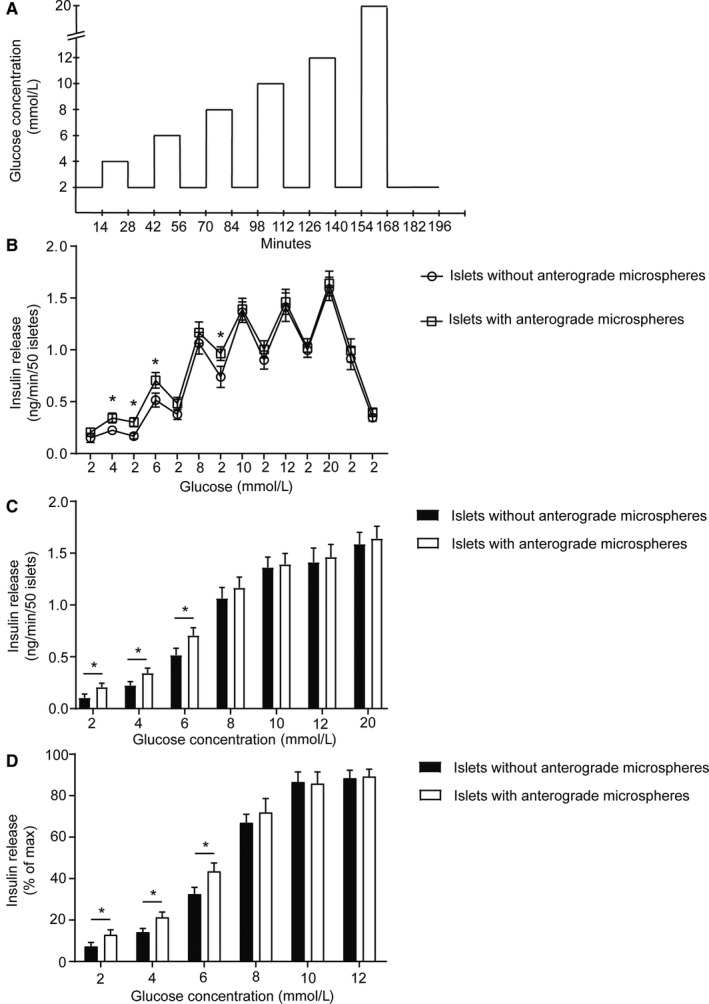
Highly blood perfused rat islets have a lower glucose threshold for insulin release than other islets. (a) Schematic drawing of the islet perifusion protocol. (b–c) Highly blood perfused islets, that is, islets with anterograde microspheres, had both a better (b–c) absolute and (d) relative insulin release at low glucose levels (2, 4, and 6 mmol/l) but similar release at higher glucose levels (8, 10, 12, and 20 mmol/l) when compared with islets without microspheres. Values are given as means ± *SEM*, *n* = 7 animals, * denotes *p* < .05

Groups of 50 size‐matched islets, with or without the presence of anterogradely injected microspheres, were inserted into filter‐covered perifusion chambers (Suprafusion 1000, 6‐channel system, Brandel). The islets were perifused (200 µl/min) with KRBH supplemented with 2 mg/ml bovine serum albumin and stepwise increasing glucose concentrations. The islets were first perifused with 2 mmol/l glucose for 30 min to acquire a baseline secretion. Perifusion was then performed with the following glucose concentrations: 2, 4, 2, 6, 2, 8, 2, 10, 2, 12, 2, 20, 2, and 2 mmol/l glucose for 14 min per glucose concentration (Figure 2a). Released insulin was analyzed by a Rat Insulin ELISA kit (Mercodia).

### 2.8 Susceptibility of islets of BB rats to immune cell infiltration in the prediabetic phase

**FIGURE 3:**
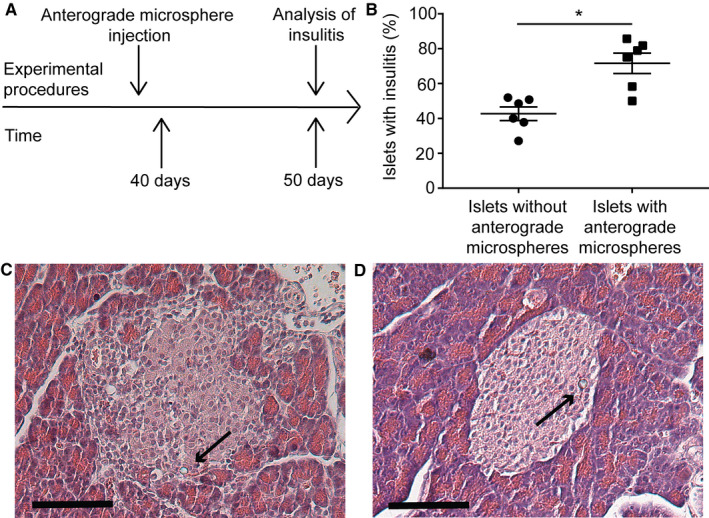
Highly blood perfused islets have a higher susceptibility to immune cell infiltration than other islets in the prediabetic BioBreeding (BB) rat. (a) Schematic drawing of the experimental set up. (b) At 50 days of age, highly blood perfused islets (as identified by anterograde microsphere injection) had an increased percentage of islets showing insulitis when compared with other islets. (c–d) Representative images of (c) highly blood perfused islet with microsphere content and insulitis from pancreas of 50‐day‐old diabetes‐prone BBrat and (d) islet with microsphere content absent of insulitis from pancreas of 50‐day‐old diabetes‐resistant BB rat; scale bar 100 µm. Arrows denote microspheres. Values are given as means ± *SEM*, *n* = 6 animals, * denotes *p* < .05

Less than 40‐day‐old prediabetic diabetes‐prone (DR.lyp/lyp) and diabetes‐resistant (DR.+/+ and DR.lyp/+) BB rats were sedated with an i.p. injection of 60 mg/kg body weight of pentobarbital sodium (Apoteket). 1.2 × 10^5^ colored microspheres were administered by anterograde injection into the ascending aorta as described earlier. After injection, the blood flow in the left carotid artery was obstructed by sutures above and below of the injection site to prevent bleeding. When 50 days old, the animals were killed and the pancreas removed, formalin‐fixed and paraffin‐embedded for histological analysis (Figure 3a). Microspheres were identified in 10‐µm‐thick hematoxylin–eosin‐stained pancreatic sections in a microscope with bright‐field illumination. Insulitis was defined semi‐quantitatively as immune infiltration into islets with mononuclear cells, using the grades 3–4 (grades 1–2 only describe infiltration into exocrine tissue) of a previously published protocol (Fuller et al., 2009). Presence or not of insulitis was evaluated in 594 ± 174 (range 167–1195) islets per animal (*n* = 6). The expression of vascular cell adhesion molecule 1 (VCAM‐1) and intercellular adhesion molecule‐1 (ICAM‐1) in microsphere‐containing islets of diabetes‐prone (with insulitis) and diabetes‐resistant rats was investigated by immunohistochemistry using mouse anti‐VCAM‐1 (1:200, Novus Biologicals), and rabbit anti‐ICAM‐1 (1:200, Abcam), respectively. Endothelial cells were stained for by a goat anti‐CD34 antibody (1:100, R&D Systems).

### 2.9 Human subjects

All procedures were approved by the human regional ethical board in Uppsala and conducted according to the declaration of Helsinki. Human islets were obtained from the Nordic Network for Islet Transplantation. Islets were isolated from three brain‐dead donors (see Table S1 for donor characteristics). From each preparation, 74–95 islets were investigated individually for their glucose threshold for insulin release. The islets were incubated in KRBH for 30 min at each of the following glucose levels: 2, 4, 6, 8, and 20 mmol/l. The supernatants were analyzed by an Ultrasensitive Human Insulin ELISA (Mercodia). Glucose threshold was defined as an increment in insulin concentration >50% when compared with that in the supernatant after incubation at 2 mmol/l glucose.

For the glucose‐clamp studies described below, all subjects were provided with oral and written information and a written informed consent was obtained from all subjects before inclusion. Inclusion criteria for the individuals with type 1 diabetes, all recruited at the Uppsala University Hospital, were disease debut within three years, age 18–35 years, BMI 20–25, a fasting C‐peptide concentration > 0.07 nmol/l and normal renal function (P‐creatinine < 100 µmol/l). Inclusion criteria for age‐ and gender‐matched healthy volunteers were normal fasting plasma glucose concentration (<6.1 mmol/l), normal HbA1C (<6%; <43 mmol/mol), BMI 20–25, and normal renal function (P‐creatinine < 100 µmol/l). Healthy volunteers were recruited by advertising.

### 2.10 Clamp at different blood glucose concentrations

All participants were fasting overnight prior to the glucose load. Participants with type 1 diabetes withdrew all insulin treatment in the morning of the experiment. Insulin (Humalog 100 IE/ml, Eli Lilly Sweden AB) and glucose (100 mg/ml) was administered through a catheter in the left cubital vein. An additional catheter was inserted into the right cubital vein to enable continuous blood sampling. Plasma glucose measurements were taken every fifth minute from clamp start. Insulin with an infusion rate of 0.040 IE/min × m^2^ was given to obtain the first glucose level of 4 mmol/l. Body surface area was calculated by the DuBois method (body surface area = 0.20247 × height (m)^0.725^ × weight (kg)^0.425^). For subjects with diabetes insulin was then administered periodically throughout the clamp study to maintain steady glucose levels. Glucose infusion was individually adjusted to clamp the participants for 15–30 min at each of the following blood glucose levels; 4, 6, 8, 10, and 12 mmol/l (±10%). At each glucose plateau, blood sampling was performed at 0 and 15–30 min (Figure 5a). After the final blood sample was obtained the individuals with diabetes continued short acting insulin treatment and all subjects were monitored after the procedure until they maintained stable normal plasma glucose levels. Pulse and oxygen saturation was monitored throughout the study.

### 2.11 Statistics

All values are given as means ± *SEM*. Paired or unpaired Student's *t*‐test were used for parametric data, and Mann–Whitney *U*‐test for nonparametric data. *p* values < .05 were considered statistically significant.

## 3 RESULTS

### 3.1 Microspheres injected anterogradely remain in the pancreatic and islet vascular beds also when followed by a retrograde microsphere injection into the pancreas

There was no difference either in the recorded pancreatic or islet blood flow between the animals subjected to an anterograde injection only and those subjected to both an anterograde and a retrograde injection of microspheres (Figure S1a and b).

### 3.2 Preferential venous drainage of highly blood perfused islets

After anterograde‐ and retrograde injections 81.0% ± 1.2% of the pancreatic islets were deficient of microspheres. 14.9% ± 1.8% of the islets were marked by microspheres (green) after anterograde injection, whereas 4.5% ± 0.6% of the islets were marked by the violet microspheres injected retrogradely. 2.0% ± 0.2% islets were marked by both violet and green microspheres. The likelihood of venous drainage of the highly blood perfused islets, marked with microspheres after anterograde injection, was thereby 2.8 ± 0.2 times higher than of size‐matched other islets without microspheres after the anterograde injection (Figure 1b and f; *p* < .05). A total number of 15,005 islets were investigated in eight animals (1876 ± 178 islets/animal).

### 3.3 Better glucose‐stimulated insulin release in islets with direct venous drainage than in other islets

There was no difference in insulin release at 1.67 mmol/l glucose between islets with a direct venous drainage (as identified by retrograde injection of microspheres) and other size‐matched islets. However, when stimulating the islets in vitro with 16.7 mmol/l glucose, the islets with a direct venous drainage had a 34% higher insulin release than other islets (Figure 1g, *p* < .05 *n* = 7 animals).

### 3.4 Lower glucose threshold for insulin release in highly blood perfused islets than other islets when investigated in vitro by perifusion

In order to determine the glucose threshold for highly blood perfused rat islets (identified by an anterograde microsphere injection) when compared with other islets, isolated islets were perifused in vitro with stepwise increasing glucose concentrations (Figure 2a). Highly blood perfused islets (identified by an anterograde microsphere injection) had a better insulin release at low glucose levels (2, 4, and 6 mmol/l glucose), but a similar release of insulin as other islets (without microspheres) at higher glucose levels (8, 10, 12, and 20 mmol/l glucose) (Figure 2b and c). The total insulin release between 2 and 20 mmol/l glucose stimulation was therefore increased in highly blood perfused islets (with microspheres) when compared with other islets (3.0 ± 0.2 ng/islet vs. 2.6 ± 0.2 ng/islet, respectively, *p* < .05; *n* = 7 animals).

The fraction of maximum release capacity released at different glucose levels indicated an increased release capacity at low glucose levels (2, 4, and 6 mmol/l glucose) in highly blood perfused islets (with microspheres) (Figure 2d). At higher glucose concentrations (8, 10, and 12 mmol/l) there was no difference in the fraction of maximum release capacity between the groups (Figure 2d).

### 3.5 Higher susceptibility to immune cell infiltration in the highly blood perfused islets than other islets of BB rats in the prediabetic phase

When injected with microspheres anterogradely at an age of less than 40 days old, all animals were normoglycemic (diabetes prone 5.7 ± 0.2 mmol/l, *n* = 6 animals; diabetes resistant 4.6 ± 0.3, *n* = 3 animals). At day 50, all animals remained normoglycemic (diabetes prone 4.3 ± 0.2 mmol/l, *n* = 6 animals; diabetes resistant 4.5 ± 0.3, *n* = 3 animals), but ~ 40% of the islets of diabetes‐prone BB rats showed signs of insulitis. Preferentially islets with a high blood perfusion (as identified by the anterogradely injected microspheres) developed immune infiltration (Figure 3b and c). However, there was no insulitis in any of the islets, including in those with microspheres, in diabetes‐resistant BB rats (Figure 3d). There was no VCAM‐1 expression in islets of either diabetes‐prone or diabetes‐resistant rats (data not shown), whereas ICAM‐1 was expressed in microsphere‐containing islets on both endothelial cells and infiltrating cells of diabetes prone, but not diabetes resistant, BB rats (for representative images, see Figure S2).

### 3.6 Individual islets from humans have differences in glucose threshold

**FIGURE 4:**
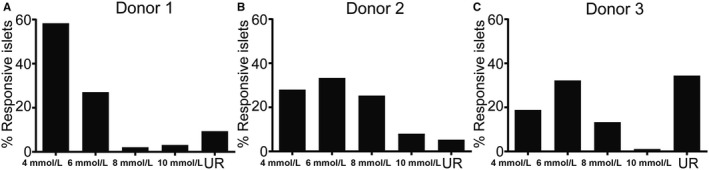
Individual islets from humans have differences in glucose threshold. Three different human islet preparations were investigated for single islet glucose threshold for insulin release (74–95 islets/preparation). In all three preparations there were some islets that responded with increased insulin release already at 4 mmol/l glucose, whereas other islets were not responsive until 6, 8, or 20 mmol/l glucose (a–c). Some islets of all three islet preparations were not responsive to glucose at all (unresponsive, UR)

Investigations of human islets from three different donors showed that the different islets from all these islet preparations varied in their glucose threshold for insulin release (Figure 4a–c). Although there were variations between the islet preparations, some islets responded already with insulin release at 4 mmol/l glucose, whereas others did not respond until stimulated with 6, 8, or even 20 mmol/l glucose. Some islets in the preparations were not responsive to glucose stimulation at all.

### 3.7 Higher glucose threshold for insulin release, when measured as C‐peptide release, in patients with type 1 diabetes when compared with healthy individuals

**FIGURE 5:**
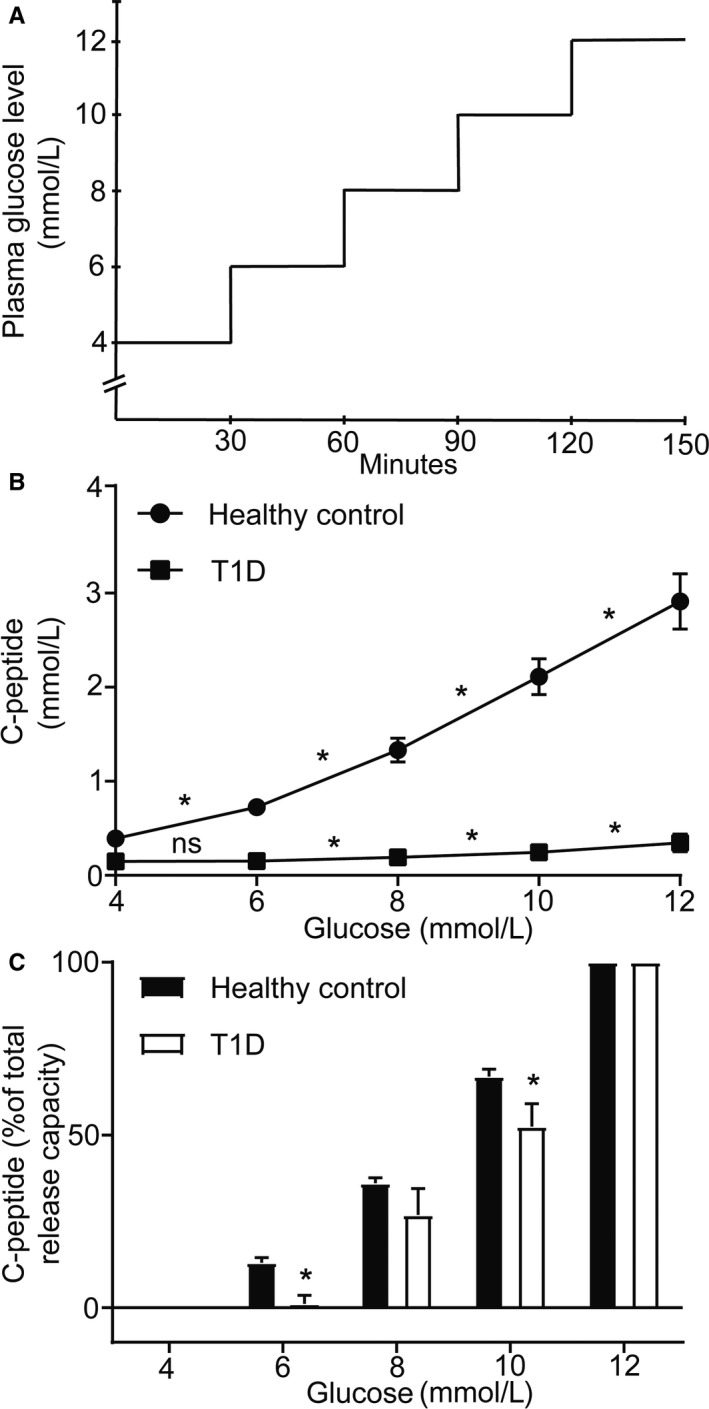
Subjects with recent onset type 1 diabetes have a higher glucose threshold than healthy controls. (a) Schematic drawing of the experimental set up. (b–c) Subjects with type 1 diabetes have an increased glucose threshold with a first increase in C‐peptide release from baseline secretion at 8 mmol/l compared to healthy subjects with an initial increase at 6 mmol/l. Values are given as means ± *SEM*, *n* = 8–10 subjects per group, * denotes *p* < .05 for increment in C‐peptide concentrations between the different blood glucose levels in (b) and for differences between healthy controls and type 1 diabetes patients in (c)

HbA1c and fasting blood glucose levels were higher in patients newly diagnosed for type 1 diabetes when compared with healthy individuals (Table 1). The subjects with type 1 diabetes had a range of fasting C‐peptide concentrations between 0.07 and 0.40 nmol/l, that is, substantially lower than the healthy controls (Table 1). The maximum C‐peptide release capacity at 12 mmol/l glucose was in the subjects with type 1 diabetes ~12% of that of healthy controls (Figure 5b). In healthy controls, a progressive increase in C‐peptide release from baseline (4 mmol/l glucose) was seen with the stepwise increase in blood glucose levels, starting at 6 mmol/l glucose. However, in subjects with type 1 diabetes, the C‐peptide release did not increase from basal levels when increasing blood glucose concentrations from 4 to 6 mmol/l glucose. An increase in C‐peptide release was seen first at blood glucose levels of 8 mmol/l and above (Figure 5c).

**TABLE 1:** Descriptive data of human subjects

	Healthy controls	Type 1 diabetes
Patients (*n*)	10	8
Gender (M/F)	(5/5)	(4/4)
Age (years)	24.5 ± 0.8	25.6 ± 1.2
BMI (kg/m^2^)	23.6 ± 1.0	23.7 ± 0.4
P‐Creatinine (µmol/l)	73.0 ± 4.5	67.4 ± 2.4
Fasting C‐peptide (nmol/l)	0.57 ± 0.07	0.22 ± 0.06*
Fasting p‐glucose (mmol/l)	5.3 ± 0.1	7.4 ± 0.6*
HbA1c [% (mmol/mol)]	4.9 ± 0.1 (30 ± 1)	6.5 ± 0.3 (48 ± 4)*

Note

Blood samples were collected after overnight fast. All data are given as means ± *SEM* unless otherwise indicated, * denotes *p* <.05.

## 4 DISCUSSION

The anatomy of islet vasculature differs between islets, where some islets have capillaries which are drained into venules emptying directly into the portal vein, where others are connected to an insulo‐acinar portal system where efferent vessels of the islets continue with the exocrine capillary network (Bonner‐Weir & Orci, 1982; Fujita, 1973; Henderson & Daniel, 1979). In this study, we have characterized a population of highly blood perfused islets and a major finding was their preferential direct venous drainage into the portal vein. This is an anatomical property which facilitates rapid delivery of insulin and other islet hormones to the liver and peripheral tissues, by surpassing drainage through the exocrine parenchyma. Previously, only large islet size has been correlated with direct venous drainage (Bonner‐Weir & Orci, 1982). By separate isolation of islets with a direct venous drainage we also observed these to have a better glucose‐stimulated insulin release in vitro than size‐matched other islets, that is, to be highly functional. Although not confirmed in this study, this finding is consistent with our previous observation, using the same technique, that highly blood perfused islets have a better glucose‐stimulated insulin release than other islets (Lau et al., 2012). In this study, we applied a perifusion technique to in more detail investigate the function of highly blood perfused islets. Despite the limitation of not including a lower glucose concentration than 2 mmol/l, these perifusion experiments indicated a lower glucose threshold for insulin release of highly blood perfused islets when compared with other islets. This opens the possibility that these islets with direct venous drainage are the major regulators of normal glucose homeostasis and only when the endocrine pancreas is substantially challenged, also other islets, mainly draining into the exocrine parenchyma, contribute with increments in insulin release. It also suggests that the previous observations of beta cells with different glucose thresholds for insulin release (Pipeleers, Kiekens, Ling, Wilikens, & Schuit, 1994; Roscioni, Migliorini, Gegg, & Lickert, 2016) are clustered to different islets and are not evenly distributed. In support of this, we have also previously identified a pool of islets with very low blood perfusion and with low metabolic activity (Olsson & Carlsson, 2011).

Worthy of note is that islet endothelium has shown tropism for coxsackie virus (Zanone et al., 2007), and that highly blood perfused islets have a denser capillary network with more endothelial cells than other islets (Lau et al., 2012). Since immune cells enter into tissues not normally through capillaries but through venules, the different anatomical organization of highly blood perfused islets by direct venous drainage may further increase their vulnerability. Indeed, there are studies indicating an increased permeability in vessels of animal models of type 1 diabetes (De Paepe, Corriveau, Tannous, Seemayer, & Colle, 1992; Jansson & Sandler, 1986; Sandler & Jansson, 1985) and also in the BB rat, this permeability defect has been suggested to be located in the veins (Majno et al., 1987). This islet venular leakage has been shown to be induced by the anti‐endothelial cell autoantibodies that occur in diabetes‐prone, but not diabetes‐resistant, BB rats (Doukas, Majno, & Mordes, 1996). Interestingly, also in type 1 diabetes individuals there is a high incidence of endothelial‐binding antibodies (Jones, Wallace, & Frier, 1992; Petty, Pottinger, Greenwood, Pearson, & Mahler, 1991; Wangel et al., 1992). If a venular defect predisposes the islets that have direct venous drainage to immune infiltration, it may be one explanation why some islets are more severely affected by insulitis than others (In't, 2011; In't Veld et al., 2007). We also show that islets of diabetes‐prone animals have an increased expression of ICAM‐1 on endothelial cells that facilitates immune cell uptake.

In order to investigate if the highly functional, highly blood perfused islets, with preferential venous drainage, are the first affected during the development of experimental type 1 diabetes, we used anterograde injection of microspheres in BB rats at age of ≤40 days for highly blood perfused islet identification. At this age, there are no signs of insulitis in the animals (Hessner et al., 2004). The experiment was thereby not confounded by inflammatory induced islet hyperperfusion. At day 50, the animals were killed and their pancreases taken to histological analysis. Approximately 40% of the islets showed signs of insulitis at 50 days of age. Interestingly, highly blood perfused islets, marked with microspheres, almost had a doubled frequency of insulitis when compared with other islets indicating a preferential infiltration of immune cells in this subpopulation of islets. By the injection of microspheres also into diabetes‐resistant animals, we could exclude that the microspheres per se induced immune infiltration; these animals showed no signs of infiltration in any of the islets. We have previously showed that there is also no increase in cellular death surrounding the microspheres acutely after microsphere injection (Ullsten et al., 2015).

The disease pattern in type 1 diabetes is similar to the one in the BB rat, with some islets that are more severely affected by insulitis than others (In't, 2011; In't Veld et al., 2007). Due to the invasive properties of the microsphere technique, a weakness is that we could not use it to study islets and disease pathogenesis in the human pancreas. However, since we observed that the highly blood perfused rat islets were characterized by a lower glucose threshold for insulin than other islets, we decided to test the hypothesis that preferentially the insulin release to low glucose concentrations are lost early during type 1 diabetes development. We first investigated if differences in glucose threshold for insulin release of human islets exist, since that has not been reported in the literature. Indeed, although there were variations between the three investigated islet preparations, some islets responded already with insulin release at 4 mmol/l glucose, whereas others did not respond until stimulated with 6, 8, or even 20 mmol/l glucose. We then compared insulin release (measured as C‐peptide) at different blood glucose concentrations in patients newly diagnosed for type 1 diabetes (having residual insulin production) to healthy individuals. Although the healthy controls increased their C‐peptide release at a blood glucose concentration of 6 mmol/l, this response was absent in patients with type 1 diabetes and the glucose threshold for increased C‐peptide release instead set to 8 mmol/l. This experimental finding is consistent with the hypothesis that islets and beta cells with the lowest glucose threshold are lost first during the development of type 1 diabetes, but a weakness is that it does not exclude other possible explanations such as glucose desensitization of beta cells. Moreover, glucose thresholds for insulin secretion are known to differ between rats and humans. Noteworthy, however, the loss of C‐peptide response to a blood glucose concentration of 6 mmol/l was present also in two patients with HbA1c well controlled in the normal range [5.4% (36 mmol/mol) and 5.7% (39 mmol/mol)].

We conclude that highly blood perfused islets have a preferential direct drainage into the portal vein and that these islets have a lower glucose threshold for insulin release and secrete more insulin than other islets. This suggests that the glucose homeostasis, if not severely challenged, is mainly controlled by these islets and not less blood perfused islets incorporated with the exocrine capillary system. At the same time these islets are the first to be attacked by the immune system in BB rats, an animal model for type 1 diabetes. In humans recently diagnosed with type 1 diabetes, the insulin release to increments of glucose concentrations in the lower range is also the first to be lost, suggesting a coupling of attack on beta cells to their metabolic activity, although not necessarily to their blood supply. Interestingly, previous studies have shown that induced beta cell rest may be used to preserve residual insulin secretion in patients newly diagnosed with type 1 diabetes (Bjork et al., 1996; Ortqvist et al., 2004).

## CONFLICT OF INTEREST

The authors have nothing to disclose.

## AUTHOR CONTRIBUTIONS

S.U. and P‐O.C. designed study. S.U, D.E., M.Q., M.F., M.S., and P‐O.C. conducted experiments. S.U. and P‐O.C. acquired and analyzed data. S.U. and P‐O.C wrote the manuscript and the other authors reviewed and approved it for submission. P‐O.C. is the guarantor of this work and, as such, had full access to all the data in the study and takes responsibility for the integrity of the data and the accuracy of the data analysis.

## Supporting information



Table S1‐Fig S1‐S2
