# Supplementary material for: Highly blood perfused, highly metabolically active pancreatic islets may be more susceptible for immune attack
Source: Physiol Rep. 2020 Jul 3;8(13):e14444. doi: 10.14814/phy2.14444 (PMC7333349; doi:10.14814/phy2.14444)
Supplement: Supplementary file 1 — Table S1‐Fig S1‐S2 [file PHY2-8-e14444-s001.pdf]

Supplemental Table 1. Descriptive data of organ donors for human islets

**Parameter**	**Donor 1**	**Donor 2**	**Donor 3**
Age (years)	54	67	62
Gender	Male	Female	Male
BMI (kg/m^2)	42.6	25.4	27.8
HbA1c (mmol/mol (%))	37 (5.5)	Not known	37 (5.5)

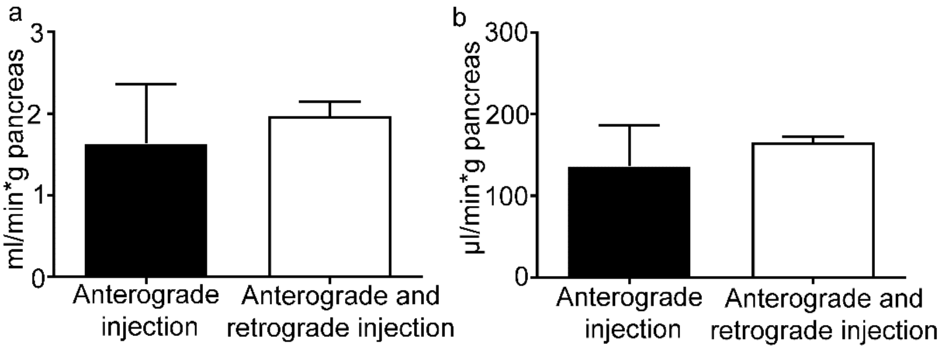

Supplemental Figure 1. Pancreatic and islet blood flow in rats given only anterograde injection or both anterograde and retrograde injection of microspheres. There were no difference in recorded (a) pancreatic blood flow or (b) islet blood flow between animals given only anterograde injection or both anterograde and retrograde injection of microspheres. Values are given as means ± SEM, n= 3 animals per group.

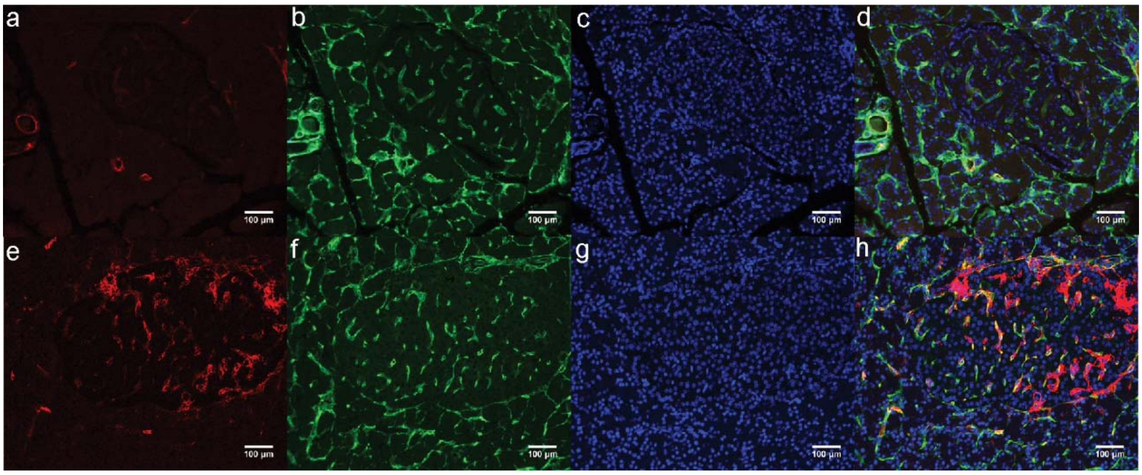

Supplemental Figure 2. Expression of intercellular adhesion molecule 1 (ICAM-1) on islet endothelial cells. (a-d) Pancreas section from diabetes-resistant (DR) BB rat showing an islet with little or no ICAM-1 expression; (a) ICAM-1 staining (red), (b) CD34 staining for endothelial cells (green), (c) Hoechst staining of cell nuclei (blue), (d) overlay of (a-c). (e-f) Pancreas section from diabetes-prone (DP) BB rat showing an islet with ICAM-1 expression on endothelial cells and infiltrating cells. (e) ICAM-1 staining (red), (f) CD34 staining for endothelial cells, (g) Hoechst staining of cell nuclei, (h) overlay of (e-g). Scale bar 100 µm.
